# Inhibition of autophagy potentiates the cytotoxicity of the irreversible FGFR1-4 inhibitor FIIN-2 on lung adenocarcinoma

**DOI:** 10.1038/s41419-022-05201-0

**Published:** 2022-08-30

**Authors:** Xiuqin Jia, Ming Xin, Juanjuan Xu, Xindong Xiang, Xuan Li, Yuhan Jiao, Lulin Wang, Jingjing Jiang, Feng Pang, Xianzhen Zhang, Jian Zhang

**Affiliations:** 1 Institute of Immunopharmaceutical Sciences, School of Pharmaceutical Sciences, Shandong University, Jinan, 250012 Shandong Province China; 2 The Key Laboratory of Molecular Pharmacology, Liaocheng People’s Hospital, Liaocheng, 252000 Shandong Province China; 3 Department of Clinical Laboratory, Liaocheng People’s Hospital, Liaocheng, 252000 Shandong Province China; 4 Department of Oncology, Liaocheng People’s Hospital, Liaocheng, 252000 Shandong Province China

**Keywords:** Pharmacology, Targeted therapies, Preclinical research

## Abstract

For patients with platinum-resistant lung adenocarcinoma (LUAD), the exploration of new effective drug candidates is urgently needed. Fibroblast growth factor receptors (FGFRs) have been identified as promising targets for LUAD therapy. The purpose of this study was to determine the exact role of the irreversible FGFR1-4 inhibitor FIIN-2 in LUAD and to clarify its underlying molecular mechanisms. Our results demonstrated that FIIN-2 significantly inhibited the proliferation, colony formation, and migration of A549 and A549/DDP cells but induced the mitochondria-mediated apoptosis of these cells. Meanwhile, FIIN-2 increased the autophagy flux of A549 and A549/DDP cells by inhibiting the mammalian target of rapamycin (mTOR) and further activating the class III PI3K complex pathway. More importantly, in vivo and in vitro experiments showed that autophagy inhibitors could enhance the cytotoxicity of FIIN-2 on A549 and A549/DDP cells, confirming that FIIN-2 induced protective autophagy. These findings indicated that FIIN-2 is a potential drug candidate for LUAD treatment, and its use in combination with autophagy inhibitors might be an efficient treatment strategy, especially for patients with cisplatin resistance.

## Introduction

Cisplatin (DDP) has been considered the cytotoxic basis of platinum-based doublet chemotherapy in lung adenocarcinoma (LUAD). However, many patients will unavoidably face treatment failure due to the emergence of platinum resistance [1]. Therefore, there is an urgent need to explore new effective drug candidates. Currently, fibroblast growth factor receptors (FGFRs) have been identified as promising targets for LUAD therapy [2]. The FGFR family comprises four members (FGFR1-4), and the frequent dysregulation of FGFRs has been revealed in LUAD using tumour sequencing technology [3–5]. This dysregulation leads to the activation of the FGFR signalling pathway, exerting a carcinogenic function [6, 7]. Therefore, it is urgent to identify the role and mechanisms of FGFR inhibitors in LUAD, especially for patients with cisplatin resistance.

According to whether covalently binding the P-loop cysteine in the ATP pocket of the FGFR intracellular tyrosine kinase domain, FGFR inhibitors are divided into reversible and irreversible FGFR inhibitors [8]. The reversible FGFR inhibitors erdafitinib and pemigatinib have been approved by the Food and Drug Administration for the treatment of metastatic urothelial carcinoma and cholangiocarcinoma, respectively [9, 10]. BGJ398 and AZD4547 have shown good efficacy in lung squamous cell carcinoma, with a disease control rate of 39-47.6% [11–13]. Irreversible FGFR inhibitors have become more promising antitumor drugs due to their better binding kinetics [14, 15]. Compared with reversible analogues, the covalent binding mode of irreversible FGFR inhibitors is less likely to dissociate from the binding site. Moreover, this inhibition process can only be reversed by protein resynthesis, so the long-term inhibition of the FGFR signalling pathway can be maintained after the drug is eliminated [16, 17]. The reported irreversible FGFR1-4 inhibitors include FIIN-2, FIIN-3 [14], futibatinib [15, 18] and PRN1371 [19]. As a representative drug of the irreversible FGFR1-4 inhibitors, FIIN-2 covalently binds to the Cys491 site of FGFR2. It shows excellent antiproliferative activity in multiple tumour cells, including lung squamous cell carcinoma H520 and large cell carcinoma H1581 [15]. However, the therapeutic efficacy of FIIN-2 has not been systematically confirmed in LUAD.

Autophagy is an intracellular process that transports damaged, degenerated or senescent proteins and organelles to lysosomes for degradation. It plays a vital role in maintaining cellular metabolism and internal environment stability. Beclin-1 binds to different molecules, such as Bcl-2 and Vps34, to form the PI3KC3-Beclin-1 complex. It plays a role in autophagosome formation and maturation [20]. After autophagy is induced, cytoplasmic LC3-I couples with the substrate PE on the membrane surface to form membrane-bound LC3-II. p62 is degraded by autolysosomes through binding to autophagy substrates. Recent studies have proven that autophagy is closely related to tumours, as it can inhibit or promote tumour development [21]. Some drugs, such as rapamycin and etoposide, exert antitumor effects by regulating autophagic cell death [22, 23]. However, most anticancer agents can induce protective autophagy in tumour cells. In this case, autophagy inhibitors can increase the cytotoxicity of anticancer drugs, and the combination of anticancer agents and autophagy inhibitors has become an excellent tumour treatment strategy [24]. Autophagy is mainly triggered by the inhibition of mammalian target of rapamycin (mTOR) or the activation of the type III PI3K complex. As a downstream of FGFR, PI3K/AKT is the most classical pathway in the regulation of mTOR inhibition and autophagy induction [25]. The irreversible FGFR1-4 inhibitor FIIN-2 can inhibit the growth of multiple tumour cells, such as gastric cancer, ovarian cancer, lung squamous cell carcinoma, and large cell carcinoma cells, by blocking PI3K/AKT signalling [14]. However, whether the antitumor effect of FIIN-2 is related to autophagy has not been reported yet.

In this study, we selected the representative human LUAD cell lines of A549 and cisplatin-resistant A549/DDP to investigate the antitumor effects of FIIN-2 and clarify the relationship between FIIN-2 and autophagy in LUAD. It provides a candidate for LUAD treatment, especially for patients with cisplatin resistance.

## Materials and methods

### Experimental reagents

FIIN-2 (#S7714) and MHY1485 (#S7811) were purchased from Selleck Chemicals (Houston, TX, USA). CQ (MB1668) and 3-MA (MB5063) were purchased from Meilunbio (Dalian, China). The primary antibodies used in this study were phospho-FGFR (p-FGFR, #3471), p-FRS2 α(#3861), p-ERK1/2 (#4370), β-actin (#3700), and GAPDH (#5174 S), and they were purchased from Cell Signalling Technologies (Danvers, MA, USA). β-tubulin (#BSM-33034) was purchased from Bioss Technology Co., Ltd. (Beijing, China). FRS2 (#11503-1-AP), Caspase-3 (#19677-1-AP), Bcl-2 (#12789-1-AP), Bax (#50599-2-Ig), MMP2 (#10373-2-AP), MMP9 (#10375-2-AP), ERK1/2 (#16443-1-AP), AKT (#10176-2-AP), p-AKT (# 66444-1-Ig), mTOR (# 66888-1-Ig), p-mTOR (#5536 T), Parkin (14060-1-AP), LC3 (#14600-1-AP), Beclin-1 (#11306-1-AP), p62 (#66184-1-1 g) and Vps34 (#12452-1-AP) were all purchased from Proteintech Group Inc. (Chicago, USA). FGFR2 (#TA503137) was purchased from Origene Technologies, Inc. (Rockville, MD, USA). The goat anti-rabbit (#ab205718) and rabbit anti-mouse (#ab205719) secondary antibodies were purchased from Abcam (Cambridge, UK). The Cell Counting Kit-8 (CCK-8) and TUNEL assay Kit (#C1088) were purchased from Beyotime Biotechnology (Shanghai, China). SDS-PAGE Gel Preparation Kit (#AR0138) was purchased from Boster Biological Technology, Ltd. (Wuhan, China). The Ki67 (#GB111141) and LC3 (#GB13431) used in immunohistochemistry were purchased from Servicebio Biological Technology Co., Ltd. (Wuhan, China).

### Immunohistochemistry

#### Pathological tissue selection

In this study, with the approval of the Medical Ethics Committee of Liaocheng People’s Hospital and informed consent from each patient or family member, we obtained 66 tissue specimens from the pathology sample bank of all patients with LUAD from June 2018 to June 2019. These archived specimens were confirmed to be lung cancer by pathological evaluation. Inclusion criteria: lung primary, complete clinical data, and not receiving antitumor therapy other than surgery. We used the 2005 World Health Organization (WHO) histological classification system to classify lung cancer. After the second evaluation ruled out samples with limited specimens, we selected 19 samples of LUAD specimens for immunohistochemistry.

#### Immunohistochemical staining

Paraffin-embedded tissue sections from 19 LUAD patients were deparaffinized in xylene, followed by hydration using an ethanol series. The antigen was restored in 0.01 M sodium citrate buffer, and endogenous peroxidase was inactivated with 10% hydrogen peroxide at 37 °C for 1 h. The specimens were incubated with 10% goat serum, followed by incubation with the primary antibody overnight. Next, the sections were added with Bio-goat anti-mouse IgG concentrate and developed with DAB (3,3'-diaminobenzidine) solution according to the SP-POD Kit (Solarbio Life Sciences Co., Ltd. Beijing, China, SP0041) instructions. Then, the sections were counterstained with haematoxylin, dehydrated with gradient ethanol, and sealed with neutral gum. Finally, two pathologists randomly selected five fields of each stained section to blindly score the staining intensity, and a third pathologist resolved any disagreements. The expression of FGFR2 in LUAD tissues was evaluated by the histochemistry score (H-score). H-score = Σ π (i + 1), where π is the proportion of stained cells (0, 1, 2, 3, 4 represent 5%, 6–25%, 26–50%, 51–75% and 75% positive cells, respectively), and i is the intensity of cell staining (0, 1, 2, 3 represent negative, weak, moderate and strong staining, respectively).

The immunohistochemical staining and H-score method of LC3, cleaved Caspase-3, and Ki67 in xenograft tumour model tissues were the same as above.

### Cell culture

The A549 and A549/DDP cell lines were purchased from Procell Life Science & Technology Co., Ltd. (Wuhan, China) and Pituo Biological Technology Co., Ltd. (Shanghai, China), respectively. The two cell lines were authenticated and confirmed to be mycoplasma-free before use. Cells were cultured in RPMI-1640 medium containing 10% foetal bovine serum (FBS, ScienCell, 0510) and penicillin–streptomycin solution (100×, Solarbio, P1400) at 37 °C and 5% CO_2_. All cells used in the experiments were in the logarithmic growth phase. A549/DDP cells were cultured in RPMI-1640 medium containing 1000 ng/mL cisplatin.

### CCK-8 cell viability and cytotoxicity assay

Cell viability was analysed by the cell counting kit-8 (CCK-8) assay. Cells were seeded in 96-well plates at a density of 6.0 × 10^3^ cells/well and then treated with different concentrations of the designated drugs for 24 h. Then, 10% CCK-8 solution was added, and the spectral absorbance at 450 nm was detected on a microplate reader.

### TUNEL staining assay to evaluate apoptosis induction

Approximately 5 × 10^5^ cells per well were seeded in 24-well plates before being exposed to different concentrations of FIIN-2 for 24 h. Cells were permeabilized with 0.3% Triton X-100 after fixing with 4% paraformaldehyde. The TUNEL assay kit was used to evaluate apoptotic cells (green staining). The number of apoptotic cells was observed with a fluorescence microscope.

### Detection of the apoptosis rate with Annexin V-fluorescein isothiocyanate (FITC)/propidium iodide (PI) staining

Apoptotic cells were detected using a FITC-Annexin V kit (BD Biosciences, 556547). Cells were treated with 0–20 μmol/L FIIN-2 for 24 h. After being transferred to a 5 ml petri dish, cells were treated with 5 μL Annexin V-FITC and 5 μL PI. Then, we incubated the mixture at 37 °C in darkness for 15 min. Then, 400 μL of 1× binding buffer was added to each tube, and the cells were analysed using a flow cytometric analysis system (FACS-Calibur, BD LSRFortessa™, USA) within 1 h. Early apoptotic cells were defined as Annexin V-FITC (+)/PI (−), while late apoptotic or necrotic cells were Annexin V-FITC (+)/PI (+).

### Detection of mitochondrial membrane potential with JC-1 staining

The mitochondrial membrane potential (MMP, ΔΨm) of cells was detected using the MMP probe JC-1 according to the protocol of the JC-1 kit (Solarbio, M8650). Briefly, cells were seeded in 24-well plates at 5 × 10^5^ per well and treated with 10 μmol/L FIIN-2 for 24 h. After adding 0.5 ml F12 medium/JC-1 working solution (1:1), the cells were incubated at 37 °C for 20 min. The fluorescence signal was detected with a fluorescence microscope (Nikon, Ti2-U). The MMP of cells was represented by the ratio of aggregated JC-1 (red)/monomeric JC-1 (green).

### Colony formation assay

Cells were seeded in 6-well plates at a density of 1 × 10^3^ cells/well and pretreated with FIIN-2 (0–5 μmol/L) when the cells adhered to the wall for 24 h. Cells were cultured for 14 days, or until most individual clones had >50 cells. The cell status was observed every 3 days and then stained with 0.1% crystal violet. After washing the cells several times with phosphate-buffered saline (PBS), we photographed each well and counted the number of cell clones.

### Wound-healing assay

Cells were seeded at a density of (2 – 5.0) × 10^5^ cells/well in 6-well plates and incubated at 37 °C until the confluence reached 90%. We scratched the cells with a 10 µL pipette tip and added the indicated drugs for treatment. Then we choose cells both sides to be uniform and recorded the 0 h data under a light microscope at 4×, 10× objective. After the cells were incubated for 24 and 48 h, we calculated the wound closure (cell migration) rate according to the following formula: wound closure (%) = (wound area at 0 h-wound area at this time point)/wound area at 0 h × 100%.

### Detection of autophagosomes with transmission electron microscopy (TEM)

After centrifugation, the cells treated with FIIN-2 were collected and fixed with TEM fixative (Servicebio, G1102). The pellet was resuspended in fixative and stored at 4 °C. Fixed cells were preembedded with 1% agarose solution and then postfixed with 1% OsO_4_ (Ted Pella, Inc.) for 2 h at 37 °C. The samples were sequentially dehydrated with graded 30–50–70–80–95–100–100% ethanol (Sinopharm Chemical Reagent Co., Ltd., 100092183) each time for 20 min and washed twice with 100% acetone (Sinopharm Chemical Reagent Co., Ltd., 10000418). Finally, the samples were embedded into EMBed 812 (embedding agent 812, SPI, 90529-77-4) and moved into a 65 °C oven to polymerize for >48 h. Ultrathin sections (60–80 nm) were prepared using an ultramicrotome (Leica, EM UC7) and stained with 2% uranium acetate and 2.6% lead citrate. Finally, the samples were observed using TEM (Hitachi, HT-7800).

### Detection of autophagy with monodansylcadaverine (MDC) staining

Cells were stained with a specific MDC autophagosome marker according to the protocol of the MDC staining kit (Solarbio, G0170-100T). Briefly, after being treated with FIIN-2 (1–20 μmol/L) for 24 h, the cells were exposed to a 10% MDC working solution for 60 min in the dark. After discarding the supernatant, the cells were fixed with anti-fluorescence quenching and observed immediately with a fluorescence microscope.

### Measurement of autophagic flux using the mRFP-GFP-LC3 lentiviral vector

Autophagic flux was analysed using the mRFP-GFP-LC3 lentiviral vector (Genechem Co., LTD. Shanghai, China). Cells transfected with the mRFP-GFP-LC3 vector (MOI 10) were seeded at 5 × 10^5^ per well in 24-well plates, followed by treatment with 10 μmol/L FIIN-2 for 24 h. After fixation with 4% paraformaldehyde, the cells were plated on coverslips. Then, the fluorescence signals of GFP and RFP were detected with confocal laser scanning microscopy (CLSM, Leica, TCS SP5). The average number of autolysosomes (GFP−/RFP+, red dots) and autophagosomes (GFP+/RFP+, yellow dots) in A549 and A549/DDP cells was calculated.

### Western blot analysis and co-immunoprecipitation (Co-IP)

Cells were collected and lysed with RIPA buffer (Beyotime, P0013C, Shanghai, China) after being treated with the indicated drugs. Then, the supernatant was collected, and the total protein concentration of the cells was determined with a BCA kit (Beyotime, P0010, Shanghai, China). After separation by 10% SDS-PAGE (Beyotime, P0014A, Shanghai, China), the samples were electrotransferred to a polyvinylidene fluoride membrane (Millipore, ISEQ00010, Billerica, MA, USA). After being treated with the specific primary antibody, the membranes were incubated with horseradish peroxidase (HRP)-conjugated secondary antibody for 1–2 h. They were washed with TBST three times. Then, the bound antibody complexes were examined by enhanced chemiluminescence solution (ECL, Amersham Pharmacia Biotech, Piscataway, NJ, USA) and analysed by a ChemiDoc XRS + system (BioRad, USA).

For co-immunoprecipitation (Co-IP) experiments, A549 and A549/DDP cells were lysed in IP Lysis/Wash Buffer (Thermo, 88805, USA). Then, 25 µL of protein A/G magnetic beads were mixed with a specific monoclonal antibody or nonspecific IgG overnight at 4 °C. The immunoprecipitated proteins were separated by SDS-PAGE and analysed by western blot analysis.

### RNA isolation and real-time quantitative PCR (RT-qPCR)

Total RNA was extracted using TRIzol (Invitrogen, 15596026), and the RNA concentration was detected with a NanodropTM 2000 spectrophotometer (Thermo Fisher, USA). cDNA was synthesized using a Beyo RTTM II First Strand cDNA Synthesis Kit (Beyotime RNase H minus, Shanghai, China), and mRNA was detected using a SYBR Green qPCR Master Mix Kit (Toroivd Technology, QST-100). The mRNA expression levels were normalized using β-actin, and the results were calculated by the 2-ΔΔCt method. The primer pairs that were used are listed in Table S1.

### Xenograft tumour model experiment

Five-week-old female nude mice (BALB/c) (~20 g body weight) were purchased from Pengyue Laboratory Animal Breeding Co., Ltd. (Jinan, China). A549 or A549/DDP cells (1 × 10^7^ cells/0.2 mL PBS/mouse) were subcutaneously injected into the right anterior axilla of nude mice. When the mouse tumours developed an average volume of 75–100 mm^3^, all nude mice with xenograft tumours were randomly divided into four groups and administered by intraperitoneal (i.p.) injection: (a) vehicle control (100 μL, saline solution); (b) CQ (30 mg/kg, qd.); (c) FIIN-2 (20 mg/kg, bid.); and (d) combination treatment of FIIN-2 (20 mg/kg, bid.) and CQ (30 mg/kg, qd.). From the first day of the intervention, we weighed the mice and measured the longest diameter (a) and the shortest diameter (b) of the tumours to calculate tumour volume (*V* = ab^2^/2) using a Vernier calliper once every 2 days. All animals were sacrificed after 28 days of consecutive treatment. The tumour tissue was removed for H&E staining, IHC, and western blot analysis. Apoptotic cells were detected using a TUNEL staining kit (Servicebio, G1501, China) containing FITC fluorescein, and the nucleus was stained blue with DAPI. The proportion of positive cells was calculated. Animal welfare and experimental procedures were carried out following the Guide for the Care and Use of Laboratory Animals (Ministry of Science and Technology of China, 2006).

### Statistical analysis

All in vitro experiments were performed at least three times, and the data were processed with SPSS V.19.0 software (IBM, Armonk, NY, USA). Quantitative data were expressed as the mean ± standard deviation (SD). Differences between groups were evaluated using a one-way analysis of variance or Student’s *t*-test. A *P*-value < 0.05 was considered statistically significant.

## Results

### The effect of FIIN-2 on FGFR-dependent signalling proteins in A549 and A549/DDP cells

**Fig. 1:**
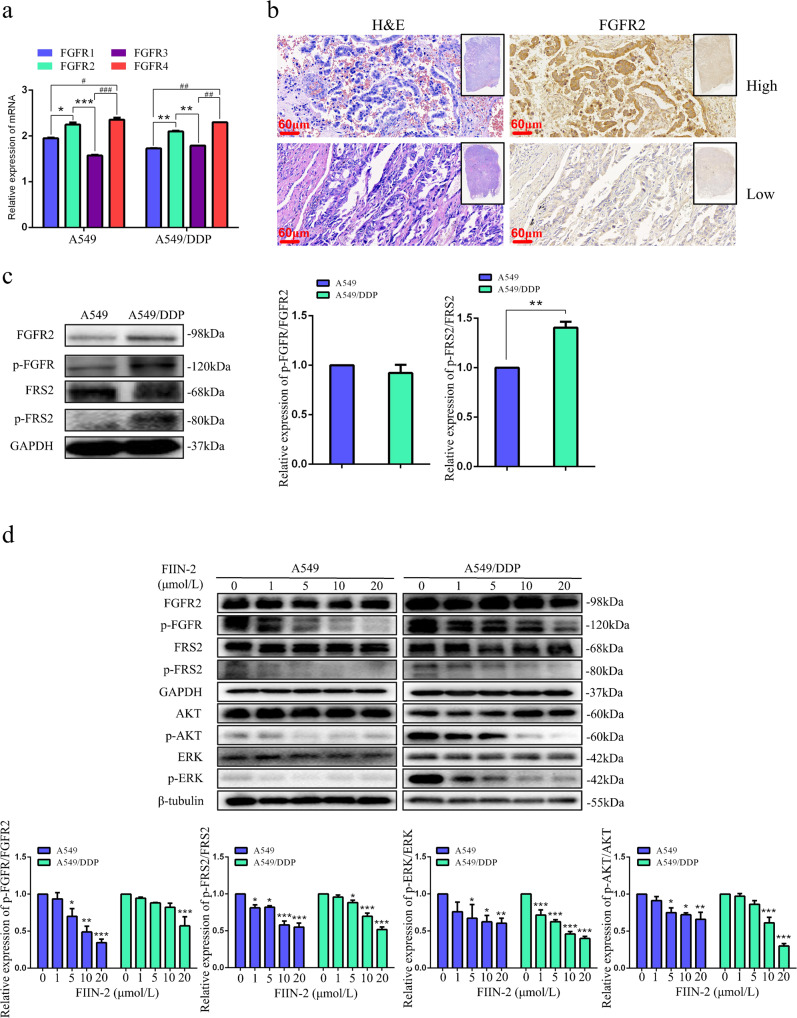
The effect of FIIN-2 on FGFR-dependent signalling proteins in A549 and A549/DDP cells. **a** The mRNA levels of FGFR1-4 in A549 and A549/DDP cells were detected by RT-qPCR. **b** Representative immunohistochemistry images of lung adenocarcinoma (LUAD) tissues with high or low expression of FGFR2. The same tissue was stained with haematoxylin and eosin (H&E) (scale bar: 60 μm, 200×). **c** The expression of FGFR2 and FGFR-dependent signalling proteins in A549 and A549/DDP cells. **d** The effects of FIIN-2 on FGFR2 and FGFR-dependent signalling proteins in A549 and A549/DDP cells were detected by western blot analysis. GAPDH and β-tubulin were used as the loading controls. Each bar corresponds to the mean ± SD of three independent experiments. *p < 0.05, **p < 0.01, ***p < 0.001; ^#^*p* < 0.05, ^##^*p* < 0.01, ^###^*p* < 0.001.

We detected the mRNA expression of FGFR1-4 in A549 and A549/DDP cells by RT-qPCR. As shown in Fig. 1a, FGFR1-4 mRNA could be detected in both cells, and the mRNA levels of FGFR2 and FGFR4 were relatively higher than those of FGFR1 and FGFR3. FGFR2 is often overexpressed in lung cancer [26, 27]. The activation of the kinase domain in FGFR2 shows high sensitivity to reversible FGFR inhibitors in vitro and in LUAD xenograft models [28, 29]. We analysed the expression of FGFR2 in LUAD tissues using immunohistochemistry, and the results confirmed that the selected 19 samples were all LUAD tissues. FGFR2 was expressed in all 19 samples (Fig. 1b), of which 9 samples (47.37%) had high FGFR2 expression (H-score: 10.67 ± 2.00) (Table S2). Activated FGFR can directly phosphorylate FGFR substrate 2α (FRS2α), a critical intracellular adaptor protein, thereby activating the downstream PI3K/AKT and MAPK/ERK pathways [6, 7]. As shown in Fig. 1c, both A549 and A549/DDP cells continuously expressed FGFR2. The expression levels of FGFR2, p-FGFR, and p-FRS2 in A549/DDP cells were all higher than those in A549 cells. These findings suggest that these proteins are differential proteins of these two cell lines and may be involved in the cisplatin resistance of A549 cells. Subsequently, we evaluated the effects of FIIN-2 on FGFR2 and FGFR-dependent signalling proteins in A549 and A549/DDP cells. As shown in Fig. 1d, after treatment with FIIN-2 for 24 h, the expression levels of p-FGFR, p-FRS2, p-AKT, and p-ERK in A549 and A549/DDP cells were reduced significantly in a dose-dependent manner. In addition, the inhibitory effect of FIIN-2 on the p-AKT signal was more evident in A549/DDP cells than in A549 cells. These results suggest that FGFR2 is involved in the occurrence and development of LUAD and that FIIN-2 can exert anti-LUAD potential by inhibiting FGFR2 signal transduction.

### FIIN-2 inhibited proliferation and induced mitochondria-mediated apoptosis in A549 and A549/DDP cells

**Fig. 2:**
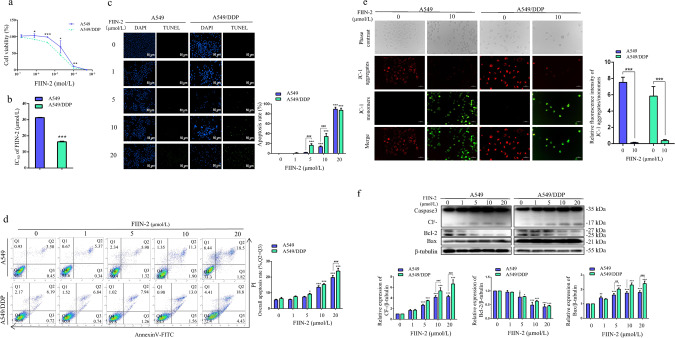
FIIN-2 inhibited proliferation and induced mitochondria-mediated apoptosis in A549 and A549/DDP cells. **a** A549 and A549/DDP cells were treated with different concentrations of FIIN-2 for 24 h, and cell viability was detected by CCK-8 assay. **b** The IC50 of FIIN-2 on A549 and A549/DDP cells was calculated. **c** TUNEL staining was used to observe the apoptosis-inducing effect of FIIN-2. **d** Cells were stained with Annexin V-fluorescein isothiocyanate (FITC) and propidium iodide (PI), and the apoptosis rate was evaluated by flow cytometry analysis. **e** Cells were treated with FIIN-2 (10 μmol/L) for 24 h, and the changes in MMP were detected by JC-1 staining (red: JC-1 aggregates; green: JC-1 monomers). Scale bar: 50 μm. **f** The effects of FIIN-2 (0–20 μmol/L) on the apoptosis-related proteins Bcl-2, Bax, and cleaved Caspase-3 were estimated by western blot analysis. β-tubulin was used as a loading control. Each bar corresponds to the mean ± SD of three independent experiments. *p < 0.05, **p < 0.01, ***p < 0.001; ^#^*p* < 0.05, ^##^*p* < 0.01, ^###^*p* < 0.001. CF-, cleaved Caspase-3.

Next, we evaluated the effects of FIIN-2 on the growth ability of human LUAD cells. A549 and A549/DDP cells were treated with different concentrations of FIIN-2 for 24 h. The cell viability was detected by CCK-8 assay, and the IC50 values were calculated. As shown in Fig. 2a, FIIN-2 reduced the viability of A549 and A549/DDP cells in a dose-dependent manner. The IC50 value of FIIN-2 in A549/DDP cells was significantly lower than that in A549 cells, these values were 16.3 ± 0.4 μmol/L and 31.3 ± 0.2 μmol/L, respectively (Fig. 2b). In addition, FIIN-2 significantly induced the apoptosis of A549 and A549/DDP cells in a dose-dependent manner (Fig. 2c, d). Decreased mitochondrial membrane potential (MMP) is a hallmark of early apoptosis. The results of JC-1 staining showed that the ratio of aggregated JC-1 (red)/monomeric JC-1 (green) was decreased in A549 and A549/DDP cells treated with FIIN-2 for 24 h compared with the control group (Fig. 2e). This result suggests that FIIN-2 may induce apoptosis through the mitochondrial pathway. Next, we examined the key indicators of the mitochondrial apoptosis pathway, Bcl-2, Bax, and the apoptosis effector protein cleaved Caspase-3. We found that FIIN-2 dose-dependently decreased the level of antiapoptotic protein Bcl-2 and increased the expression of proapoptotic proteins Bax and cleaved Caspase-3 (Fig. 2f). Notably, the FIIN-2-induced apoptosis was more pronounced in A549/DDP cells than in A549 cells (Fig. 2d, f). These results indicate that FIIN-2 can inhibit proliferation and induce mitochondria-mediated apoptosis in LUAD cells, especially in A549/DDP cells.

### FIIN-2 inhibited the colony formation and migration ability of A549 and A549/DDP cells

**Fig. 3:**
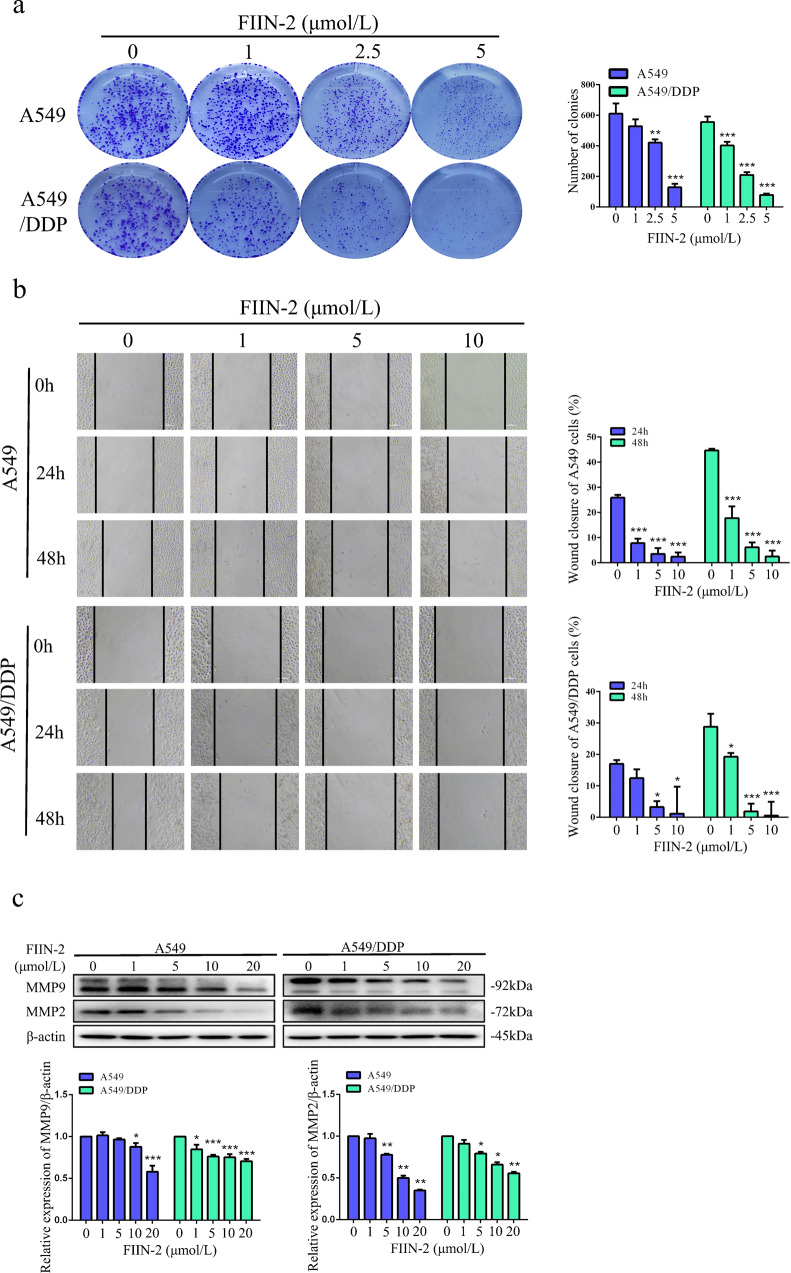
FIIN-2 inhibited the colony formation and migration ability of A549 and A549/DDP cells. **a** A549 and A549/DDP cells were pretreated with 0–5 μmol/L FIIN-2 and cultured until most individual clones exceeded 50 cells, and the colony formation of both cells was analysed. **b** The wound-healing assay was used to observe the effects of FIIN-2 (0–10 μmol/L) on cell migration. **c** Cells were treated with 0–20 μmol/L FIIN-2 for 24 h, and the levels of MMP2 and MMP9 were detected by western blot analysis. β-actin was used as a loading control. Each bar corresponds to the mean ± SD of three independent experiments. *p < 0.05, **p < 0.01, ***p < 0.001; ^#^*p* < 0.05, ^##^*p* < 0.01, ^###^*p* < 0.001.

Colony formation and migration represent the independent survival and metastasis ability of tumour cells. We observed that FIIN-2 treatment significantly reduced the colony-forming ability of both cells, and the reduction was more evident in A549/DDP cells than in A549 cells (Fig. 3a). Furthermore, the migration ability of A549 and A549/DDP cells was also significantly reduced by treatment with FIIN-2 (Fig. 3b), and the migration-related proteins MMP2 and MMP9 were inhibited (Fig. 3c). These results confirm that FIIN-2 could dramatically inhibit the migration and colony formation ability of A549 and A549/DDP cells.

### FIIN-2 induced autophagic flux in A549 and A549/DDP cells through mTOR inhibition and further activation of the class III PI3K pathway

**Fig. 4:**
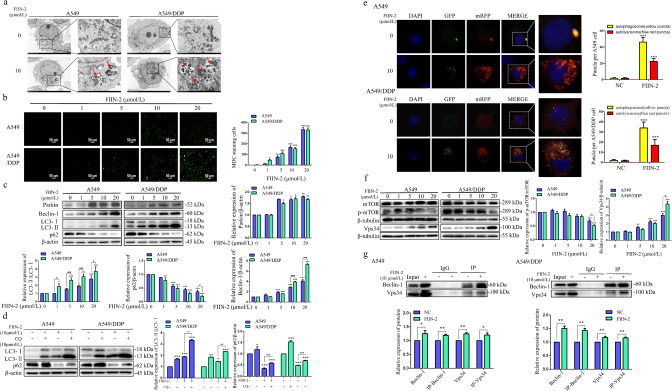
FIIN-2 induced autophagic flux in A549 and A549/DDP cells through mTOR inhibition and the activation of the class III PI3K pathway. **a** A549 and A549/DDP cells were treated with 10 μmol/L FIIN-2 for 24 h, and the autophagy characteristics were observed by transmission electron microscopy (TEM). **b** The MDC assay was used to observe the induction of autophagy by FIIN-2 (0–20 μmol/L) in A549 and A549/DDP cells. **c** Cells were treated with FIIN-2 (0–20 μmol/L) for 24 h, and the levels of Parkin, Beclin-1, p62, and LC3 were detected by western blot analysis. **d** A549 and A549/DDP cells were cotreated with 10 μmol/L CQ and 10 μmol/L FIIN-2 for 24 h, then the levels of LC3-II/LC3-I and p62 were detected by western blot. **e** After the mRFP-GFP-LC3 lentiviral vector was transfected into A549 and A549/DDP cells, the effect of FIIN-2 (10 μmol/L) on autophagic flux was evaluated (yellow dots: autophagosomes; red dots: autophagolysosomes). **f** Cells were treated with FIIN-2 (0–20 μmol/L) for 24 h, and the levels of p-mTOR and Vps34 were detected by western blot analysis. **g** The effect of FIIN-2 (10 μmol/L) on the Vps34-Beclin-1 complex was analysed by immunoprecipitation. β-actin and β-tubulin were used as the loading controls. Each bar corresponds to the mean ± SD of three independent experiments. *p < 0.05, **p < 0.01, ***p < 0.001; ^#^*p* < 0.05, ^##^*p* < 0.01, ^###^*p* < 0.001.

Autophagy plays a vital role in tumorigenesis and development. As a downstream of FGFR signalling, the PI3K/AKT/mTOR pathway is a classic autophagy regulatory pathway [25]. Therefore, we investigated whether the antitumor effect of FIIN-2 is relevant to autophagy. As the transmission electron microscopy (TEM) results showed, the number of autolysosomes in A549 and A549/DDP cells treated with FIIN-2 was significantly increased (Fig. 4a). We also observed that FIIN-2 dose-dependently increased the number of autophagosomes using MDC staining in A549 and A549/DDP cells (Fig. 4b). The mitochondrial damage caused by decreased MMP will activate the function of Parkin, allowing damaged mitochondria to be degraded by the autophagic pathway [30]. When cells were treated with FIIN-2, the expression of Parkin, Beclin-1 and the accumulation of LC3-II increased, whereas the expression of p62 decreased (Fig. 4c). Notably, FIIN-2-induced changes were more evident in A549/DDP cells than in A549 cells. These results indicate that FIIN-2 could dose-dependently induce autophagy in A549 and A549/DDP cells.

The increase of autophagosome formation and/or autophagy flux blockade can lead to the accumulation of LC3-II. To further confirm the effect of FIIN-2 on autophagic flux, chloroquine (CQ) was used to prevent autophagolysosome degradation. The combination of FIIN-2 and CQ further enhanced LC3-II accumulation and p62 expression compared to FIIN-2 alone (Fig. 4d). In addition, the influence of FIIN-2 on autophagic flux was further verified in A549 and A549/DDP cells stably expressing mRFP-GFP-LC3. As shown in Fig. 4e, the number of autophagosomes (yellow dots) and autophagolysosomes (red dots) in the FIIN-2-treated cells was increased in a concentration-dependent manner. These data confirm that FIIN-2 can induce autophagic flux in A549 and A549/DDP cells.

Autophagy induction is mainly triggered by mTOR inhibition and the activation of the type III PI3K pathway. The type III PI3K pathway contains only one member, PI3KC3 (Vps34), that forms the PI3KC3/Vps34-Beclin-1 complex [25]. To explore the molecular mechanism by which FIIN-2 induces autophagy, we detected the effect of FIIN-2 on mTOR and Vps34. The results showed that FIIN-2 inhibited the level of p-mTOR and upregulated the expression of Vps34 (Fig. 4f). These effects were more pronounced in A549/DDP cells than in A549 cells. Furthermore, immunoprecipitation analysis showed that FIIN-2 could recruit more Vps34 as Beclin-1 expression increased (Fig. 4g). These results indicate that FIIN-2 could induce autophagy in LUAD by inhibiting mTOR and activating the Beclin-1-Vps34 complex. As a core regulator of autophagy, mTOR inhibition can initiate autophagy in tumour cells by activating the ULK1 complex and further activating the type III PI3K complex [31, 32]. To investigate whether FIIN-2-induced autophagy could be caused by mTOR inhibition-mediated activation of the type III PI3K complex, we activated the mTOR pathway with the mTOR activator MHY1485. As shown in Fig. S1, compared with FIIN-2 alone, the combination of FIIN-2 and MHY1485 significantly increased p-mTOR (Ser2448) levels and decreased Beclin-1, Vps34 expression and LC3 accumulation (Fig. S1). These findings indicate that the activation of the mTOR pathway attenuated FIIN-2-induced upregulation of Beclin-1, Vps34 and induction of autophagy, suggesting that FIIN-2 could induce autophagy by mTOR inhibition and further activation of the class III PI3K pathway.

### Inhibition of autophagy enhanced FIIN-2-mediated cytotoxicity in A549 and A549/DDP cells

**Fig. 5:**
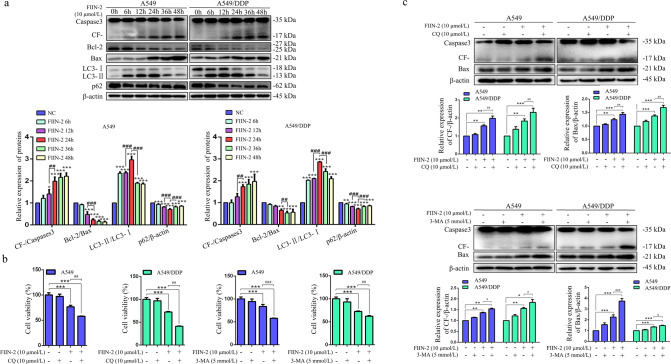
Inhibition of autophagy enhanced FIIN-2-mediated cytotoxicity in A549 and A549/DDP cells. **a** Cells were treated with FIIN-2 (10 μmol/L) for 48 h, and the levels of Bcl-2, Bax, cleaved Caspase-3, LC3, and p62 at different times were detected by western blot analysis. Cells were treated with FIIN-2 (10 μmol/L), CQ (10 μmol/L), or 3-MA (5 mmol/L) alone and FIIN-2 combined with CQ/3-MA for 24 h. **b** Cell viability was detected by CCK-8 assay. **c** The expression of Bax and cleaved Caspase-3 was detected by western blot analysis. β-actin was used as a loading control. Each bar corresponds to the mean ± SD of three independent experiments. *p < 0.05, **p < 0.01, ***p < 0.001; ^#^*p* < 0.05, ^##^*p* < 0.01, ^###^*p* < 0.001. CF-, cleaved Caspase-3.

Crosstalk between autophagy and apoptosis exists in many human tumour cells [33]. To elucidate the relationship between autophagy and apoptosis caused by FIIN-2, we investigated the changes of autophagy- and apoptosis-related proteins at different times after FIIN-2 treatment. The expression levels of cleaved Caspase-3 and LC3-II were upregulated and peaked at 24 h, accompanied by the downregulation of Bcl-2/Bax and p62 compared with the control group (Fig. 5a). These findings indicate that FIIN-2 could induce autophagy and apoptosis in LUAD cells. However, FIIN-2-induced apoptosis lasted for up to 48 h, while autophagy decreased after 24 h of treatment with FIIN-2. To further determine the effect of FIIN-2-induced autophagy on the apoptosis of LUAD cells, the late autophagy inhibitor CQ or the early autophagy inhibitor 3-methyladenine (3-MA) was used to treat cells alone or together with FIIN-2. The results showed that neither CQ nor 3-MA alone could affect the viability of A549 and A549/DDP cells. However, FIIN-2 combined with CQ or 3-MA showed a stronger inhibitory effect on the viability of A549 and A549/DDP cells than FIIN-2 alone (Fig. 5b). Furthermore, cotreatment with FIIN-2 and CQ/3-MA also significantly increased the expression of the proapoptotic proteins Bax and cleaved Caspase-3 (Fig. 5c). These results indicate that FIIN-2 induced protective autophagy and inhibiting autophagy enhance FIIN-2-mediated cytotoxicity in LUAD cells.

### Combined treatment with autophagy inhibitors augmented the anti-LUAD effect of FIIN-2 in vivo

**Fig. 6:**
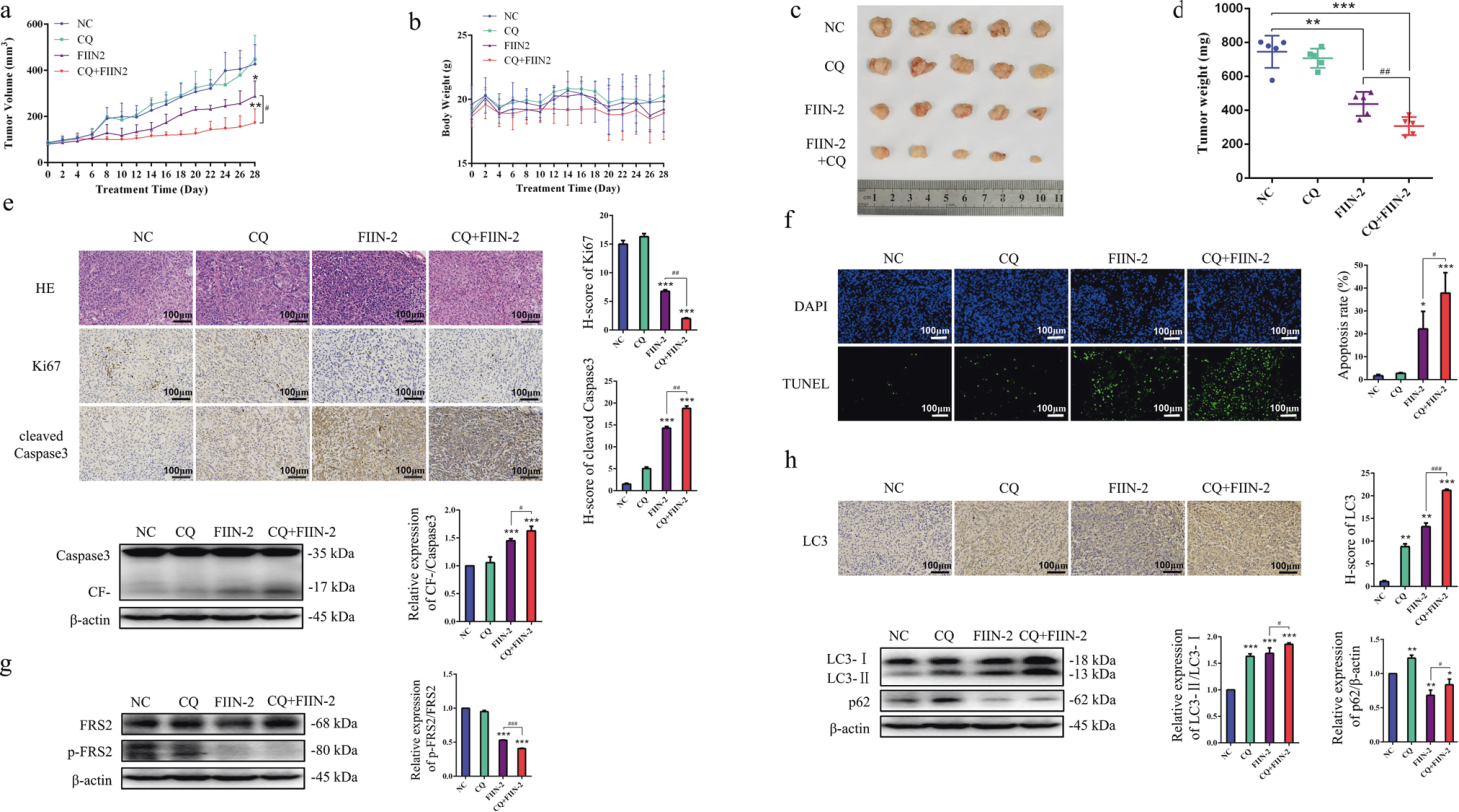
The anti-LUAD effect of FIIN-2 alone and combined with CQ in xenograft models derived from A549/DDP cells. The nude mouse xenograft model derived from A549/DDP cells was established. They were treated with vehicle control (100 μL saline solution, qd.), CQ (30 mg/kg, qd.), FIIN-2 (20 mg/kg, bid.), or FIIN-2 (20 mg/kg, bid.) combined with CQ (30 mg/kg, qd.) via intraperitoneal injection for 28 consecutive days. The tumour volume (**a**) and body weight (**b**) of the mice were measured every other day. **c** Tumours were removed and imaged. **d** The tumour weight of each group was calculated. **e** Immunohistochemical staining and/or western blot analysis were performed to detect the expression of the proliferation marker Ki67 and proapoptotic molecule cleaved Caspase-3. **f** A TUNEL assay was used to detect cell apoptosis. **g** The expression of p-FRS2 was detected by western blot analysis. **h** The expression of the autophagy markers LC3 and p62 was detected by immunohistochemical staining and/or western blot analysis. Magnification 200×, scale bars: 100 μm. Each measurement is an average ± SD (*n* = 5). *p < 0.05, **p < 0.01, ***p < 0.001; ^#^*p* < 0.05, ^##^*p* < 0.01, ^###^*p* < 0.001.

To evaluate the in vivo anti-LUAD effect of FIIN-2 combined with autophagy inhibitors, nude mice were subcutaneously inoculated with A549/DDP (Fig. 6) and A549 (Fig. S2) cells. When the tumour volume reached 75–100 mm^3^, these mice were randomly divided into the control group, CQ group, FIIN-2 group, and CQ combined with FIIN-2 group and treated for 28 consecutive days. Compared with the control group, CQ alone did not significantly influence tumour growth. However, FIIN-2 alone exhibited a significant suppressive effect on tumour growth, which could be enhanced by combination use with CQ (Figs. 6a and S2a). The body weight of mice in each group did not significantly decrease (Figs. 6b, S2b). In addition, the volume and weight of the tumours from FIIN-2-treated mice were significantly reduced compared to control or CQ-treated mice, which was strengthened by the combination with CQ treatment (Figs. 6c, d, and S2c, d). H&E staining confirmed that the two constructed xenograft tumour models were LUAD (Figs. 6e and S2e). The immunohistochemical analysis showed that FIIN-2 treatment could inhibit the expression of Ki67 compared to the control and CQ-treated groups, and FIIN-2 combined with CQ inhibited Ki67 more robustly than FIIN-2 alone (Figs. 6e and S2e). TUNEL staining showed that the apoptosis rate of tumour cells in the FIIN-2 group was significantly increased compared with the control and CQ groups, which was further increased in the FIIN-2 combined with CQ group (Figs. 6f and S2f), accompanied by the upregulation of cleaved Caspase-3 (Figs. 6e, S2e).

Consistently, we also observed the inhibitory effect of FIIN-2 on the FGFR downstream substrate p-FRS2 (Figs. 6g and S2g). The LC3 levels were increased, and the p62 levels were decreased compared to the control and CQ groups (Figs. 6h and S2h), which further augmented by the combination of CQ. These results indicated that FIIN-2 could inhibit LUAD growth in vivo and that blocking autophagy could further enhance the cytotoxicity of FIIN-2.

## Discussion

Upon ligand binding, FGFRs autophosphorylate and recruit the critical intracellular adaptor protein FRS2α, leading to the activation of the PI3K/AKT/mTOR and MAPK/ERK pathways [7]. Aberrant activation of FGFR signalling has been associated with cancer cell proliferation, apoptosis, migration, and platinum resistance in a variety of solid tumours [2, 34]. Therefore, FGFR inhibitors are expected to be a new therapeutic option for platinum-resistant LUAD. Here, we found that the irreversible FGFR inhibitor FIIN-2 inhibited FGFR downstream of p-FRS2 and the AKT and ERK pathways, which is consistent with the results of Tan et al. in Ba/F3 cells mutated at the V564 site of FGFR2 [14]. FIIN-2 had a definite antitumor effect on A549 cells. Furthermore, its ability to inhibit proliferation, clone formation and induce apoptosis was more pronounced in A549/DDP cells. We speculate that this is related to the activation of downstream FRS2 signalling due to the high expression of FGFR2 in A549/DDP cells. Our in vivo experiments also confirmed that FIIN-2 suppressed p-FRS2 signalling, which is consistent with the reversible FGFR inhibitor BGJ-398 in the LUAD mouse model driven by mutations in the FGFR2 kinase domain [28]. Therefore, FGFR2 is related to the occurrence and development of LUAD, and FIIN-2 can exert an anti-LUAD effect by inhibiting p-FRS2 and its downstream AKT and ERK pathways, especially in cisplatin-resistant LUAD.

Apoptosis is a key indicator of tumour targeted therapy [35]. Here, we found that FIIN-2 could induce apoptosis in A549 and A549/DDP cells in vitro and in vivo, and FIIN-2-induced apoptosis was more pronounced in A549/DDP cells than in A549 cells. Decreased MMP is a hallmark of early apoptosis [36]. We observed that the MMP of A549 and A549/DDP cells decreased after FIIN-2 treatment, suggesting that FIIN-2 may induce apoptosis through the mitochondrial pathway. The Bcl-2 and Bax proteins in the Bcl-2 family play key regulatory roles in the mitochondrial apoptosis pathway [37]. We found that FIIN-2 dose- and time-dependently downregulated Bcl-2 levels and upregulated Bax levels, which activated the apoptosis effector protein Caspase-3 to induce apoptosis in A549 and A549/DDP cells. These findings further confirm that FIIN-2 initiates the apoptotic cascade of the mitochondrial pathway in LUAD cells.

Autophagy is a conservative cellular catabolic process [21]. During metabolic stress, such as drug treatment, tumour cells can clear and recycle various damaged organelles (e.g., mitochondria) by initiating autophagy to protect tumour cells [38]. Mitochondrial damage leads to a decrease in MMP. Then, Pink1 is recruited to the mitochondrial membrane and Parkin triggers multiple downstream signals. These signals ultimately lead to phagocytosis and degradation of damaged mitochondria by autophagosomes [30]. Here, we found that FIIN-2 upregulated Parkin, Beclin-1 and LC3-II levels but downregulated p62 expression. Moreover, FIIN-2 increased autophagic lysosomes, acidic autophagic vesicles, and autophagic flux, indicating that FIIN-2 indeed induced autophagy in LUAD. Currently, regarding the relationship between FGFR inhibitors and autophagy, only the radiosensitization of reversible FGFR inhibitor AZD4547-induced autophagy has been studied in head and neck squamous cell carcinoma and non-small-cell lung cancer [39, 40]. Our findings provide a reference for revealing the relationship between FGFR inhibitors and autophagy.

As a critical regulator of autophagy, mTOR can activate the ULK1 complex to induce autophagy under starvation or inhibited by drugs [41, 42]. The mTOR inhibition or activation of the type III PI3K complex can initiate the autophagic process of tumour cells [43]. Studies have found that the inhibition of AKT or ERK signalling can mediate mTOR inhibition to induce autophagy [44, 45]. Here, we found that FIIN-2 reduced the phosphorylation levels of AKT, ERK and mTOR, indicating that FIIN-2-induced autophagy was associated with mTOR inhibition mediated by AKT and ERK signalling inhibition. As a scaffold protein of the type III PI3K complex, Beclin-1 can promote the localization of autophagy proteins to autophagic vesicles. Vps34 is essential for the recruitment of autophagy-related proteins to autophagic vesicles [46]. The type III PI3K pathway can be positively regulated by Beclin-1 and negatively regulated by Bcl-2 [25]. We found that FIIN-2 increased Beclin-1 levels and reduced Bcl-2 levels in A549 and A549/DDP cells. Therefore, we speculate that the autophagy induced by FIIN-2 may also be related to the activation of the type III PI3K pathway. Subsequently, our Co-IP experimental results confirmed that as Beclin-1 expression increased, the amount of recruited Vps34 was increased by FIIN-2 treatment. These results indicate that the autophagy induced by FIIN-2 was not only caused by mTOR inhibition but also type III PI3K complex activation. Using the mTOR activator MHY1485, we confirmed that FIIN-2 could induce autophagy by mTOR inhibition and further activation of the class III PI3K pathway. Furthermore, the autophagy induced by FIIN-2 in A549/DDP cells was significantly increase compared with that in A549 cells, which may be related to the more pronounced mTOR inhibition and Vps34 upregulation in A549/DDP cells.

Autophagy has a dual role in inducing tumour cell death and survival [47], with its specific effects depending on survival conditions and tumour characteristics [48]. Autophagy excessively degrades the cytoplasm, directly leading to autophagic cell death or participating in inducing apoptosis [49]. However, autophagy activation can play an anti-apoptotic role by degrading and recycling cytoplasmic components such as damaged mitochondria [33]. Decreased MMP can lead to mitochondrial damage and activate Parkin, resulting in the degradation of damaged mitochondria by the autophagy pathway [30]. Here, we found that MMP and Bcl-2/Bax expression were decreased in A549 and A549/DDP cells after FIIN-2 treatment, while Parkin, the bridge protein linking autophagy and apoptosis, was increased. However, FIIN-2-induced apoptosis lasted for up to 48 h, while autophagy decreased after 24 h of treatment with FIIN-2. These data suggest that FIIN-2 can induce both apoptosis and autophagy at the early stage of administration via mitochondrial damage, but FIIN-2-induced apoptosis can last for a longer time than autophagy. Thus, it can overcome protective autophagy, resulting in LUAD cell death. Therefore, the combination of the autophagy inhibitor 3-MA or CQ, the anti-proliferative and proapoptotic effects of FIIN-2 are enhanced, as confirmed by our in vitro and in vivo results.

**Fig. 7:**
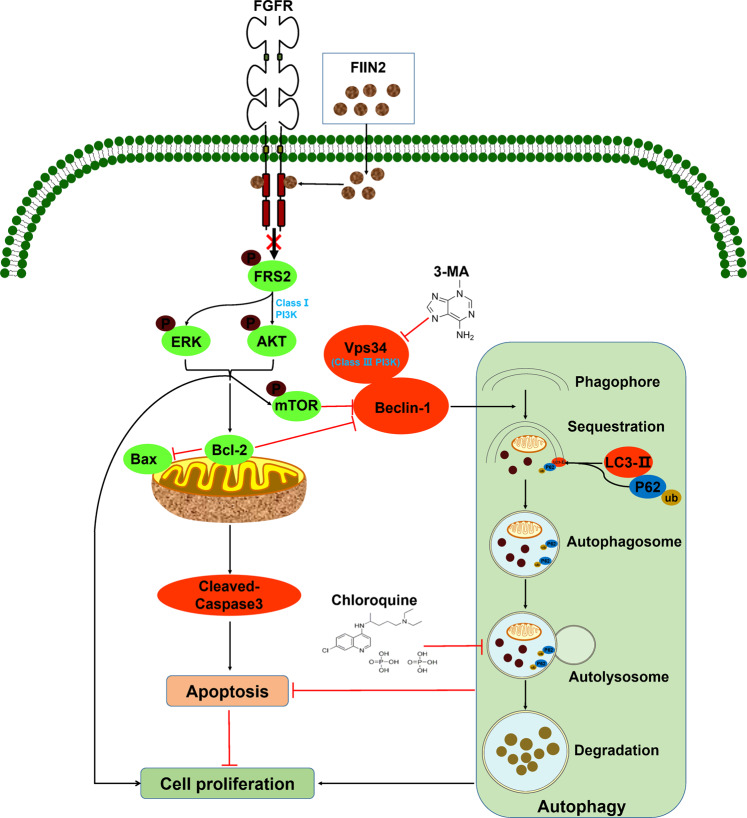
The potential regulatory mechanism of inhibiting autophagy to enhance the cytotoxicity of FIIN-2 on A549 and A549/DDP cells. FIIN-2 can inhibit proliferation and induce mitochondria-mediated apoptosis in A549 and A549/DDP cells by inhibiting FRS2 and its downstream AKT and ERK pathways. FIIN-2 can also induce protective autophagy by mTOR inhibition and further activation of the class III PI3K pathway (Vps34-Beclin-1 complex). Moreover, cotreatment of FIIN-2 with the early stage or late-stage autophagy inhibitors 3-MA or CQ enhanced the cytotoxicity of FIIN-2 by inhibiting proliferation and inducing apoptosis in A549 and A549/DDP cells.

In conclusion, we demonstrated that FIIN-2 exhibited significant antitumor effects on LUAD by inducing mitochondrial damage-mediated apoptosis. Moreover, FIIN-2 induced protective autophagy through mTOR inhibition and further class III PI3K complex activation. More importantly, autophagy inhibitors enhanced the cytotoxicity of FIIN-2 to LUAD in vitro and in vivo (Fig. 7). Therefore, FIIN-2 is expected to be a drug candidate for LUAD, and autophagy inhibitors can be used as sensitizers to enhance the antitumor effect of FIIN-2, especially for patients with cisplatin resistance.

## Supplementary information

Supplemental Figure Legends

Supplemental Figure S1.

Supplemental Figure S2.

Supplemental Table S1.

Supplemental Table S2.

Original full length western blots

Reproducibility Checklist

## Data Availability

The data used to support the findings of this study are available from the corresponding author upon request.
