# Supplementary material for: Inhibition of autophagy potentiates the cytotoxicity of the irreversible FGFR1-4 inhibitor FIIN-2 on lung adenocarcinoma
Source: Cell Death Dis. 2022 Aug 30;13(8):750. doi: 10.1038/s41419-022-05201-0 (PMC9428205; doi:10.1038/s41419-022-05201-0)
Supplement: Supplementary file 1 — Supplemental Figure Legends [file 41419_2022_5201_MOESM1_ESM.docx]

**Supplemental Figure Legends**

**Supplemental Figure S1.** **FIIN-2 induced autophagy through mTOR inhibition and further activation of the class III PI3K pathway.** Cells were treated with FIIN-2 (10 μmol/L) and MHY1485 (an mTOR activator) (2 μmol/L) for 24 h, and the levels of mTOR, p-mTOR, Beclin-1, Vps34 and LC3-II/LC3-I were detected by western blot analysis. β-actin was used as the loading control. Each bar corresponds to the mean ± SD of three independent experiments. **p* < 0.05, ***p* < 0.01, ****p* < 0.001; ^#^*p* < 0.05, ^##^*p* < 0.01, ^###^*p* < 0.001.

**Supplemental Figure S2. The anti-LUAD effect of FIIN-2 combined with** **CQ in xenograft models derived from A549 cells.** A nude mouse xenograft model derived from A549 cells was established. These mice were treated with vehicle control (100 μL saline solution, qd.), CQ (30 mg/kg, qd.), FIIN-2 (20 mg/kg, bid.), or FIIN-2 (20 mg/kg, bid.) combined with CQ (30 mg/kg, qd.) via intraperitoneal injection for 28 consecutive days. The tumour volume (a) and body weight (b) of the mice were measured every other day. (c) Tumours were removed and imaged. (d) The tumour weight of each group was calculated. (e) Immunohistochemical staining and/or western blot analysis were performed to detect the expression of the proliferation marker Ki67 and proapoptotic molecule cleaved Caspase-3. (f) A TUNEL assay was used to detect cell apoptosis. (g) The expression of p-FRS2 was detected by western blot analysis. (h) The expression of the autophagy markers LC3 and p62 was detected by immunohistochemical staining and/or western blot analysis. Magnification 200 ×, scale bars: 100 μm. Each measurement is an average ± SD (n=5). **p* < 0.05, ***p* < 0.01, ****p* < 0.001; ^#^*p* < 0.05, ^##^*p* < 0.01, ^###^*p* < 0.001.
