# Supplementary figures and images for: Inhibition of autophagy potentiates the cytotoxicity of the irreversible FGFR1-4 inhibitor FIIN-2 on lung adenocarcinoma
Source: Cell Death Dis. 2022 Aug 30;13(8):750. doi: 10.1038/s41419-022-05201-0 (PMC9428205; doi:10.1038/s41419-022-05201-0)

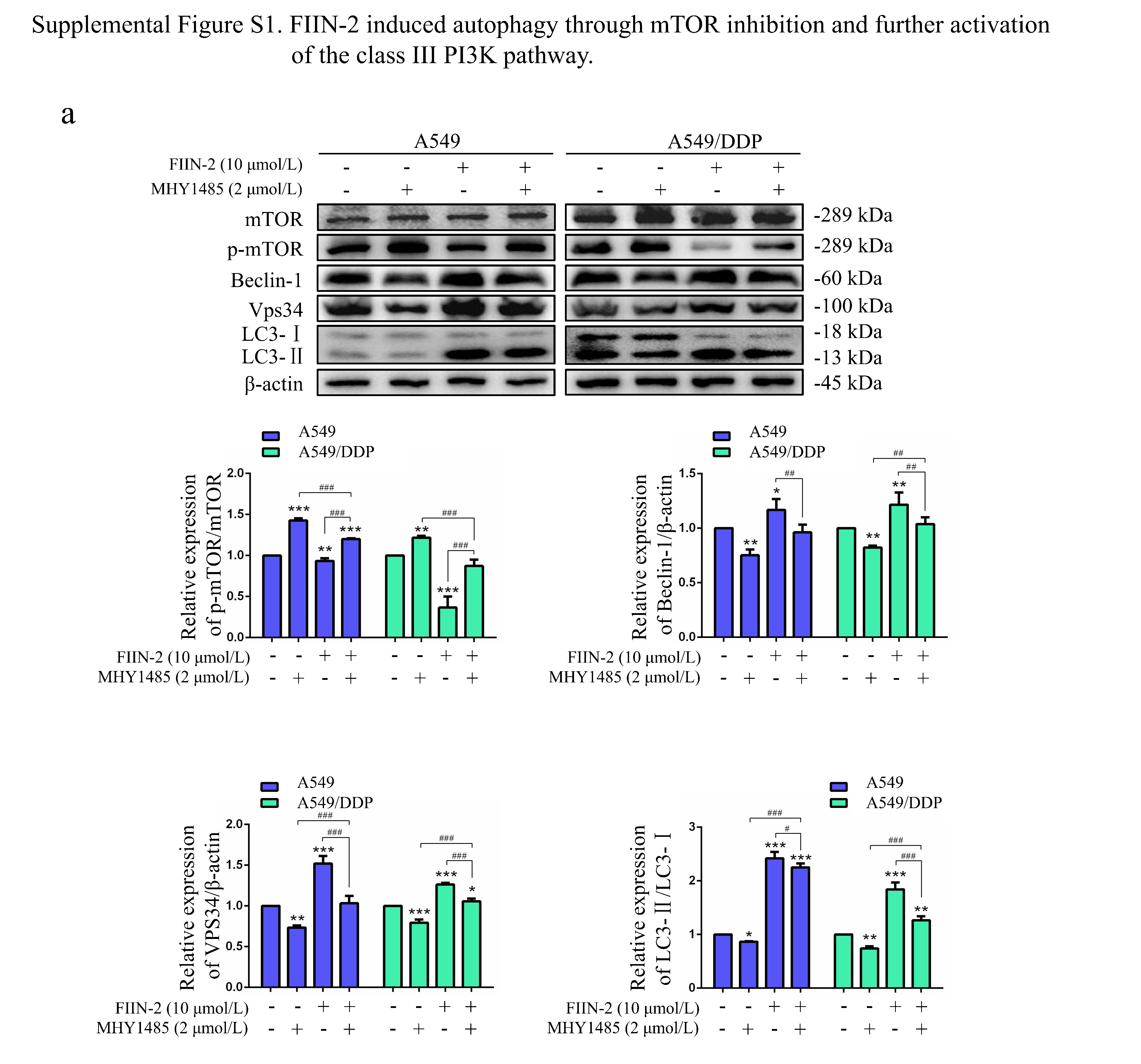

Supplement: Supplementary file 2 — Supplemental Figure S1. [file 41419_2022_5201_MOESM2_ESM.png]

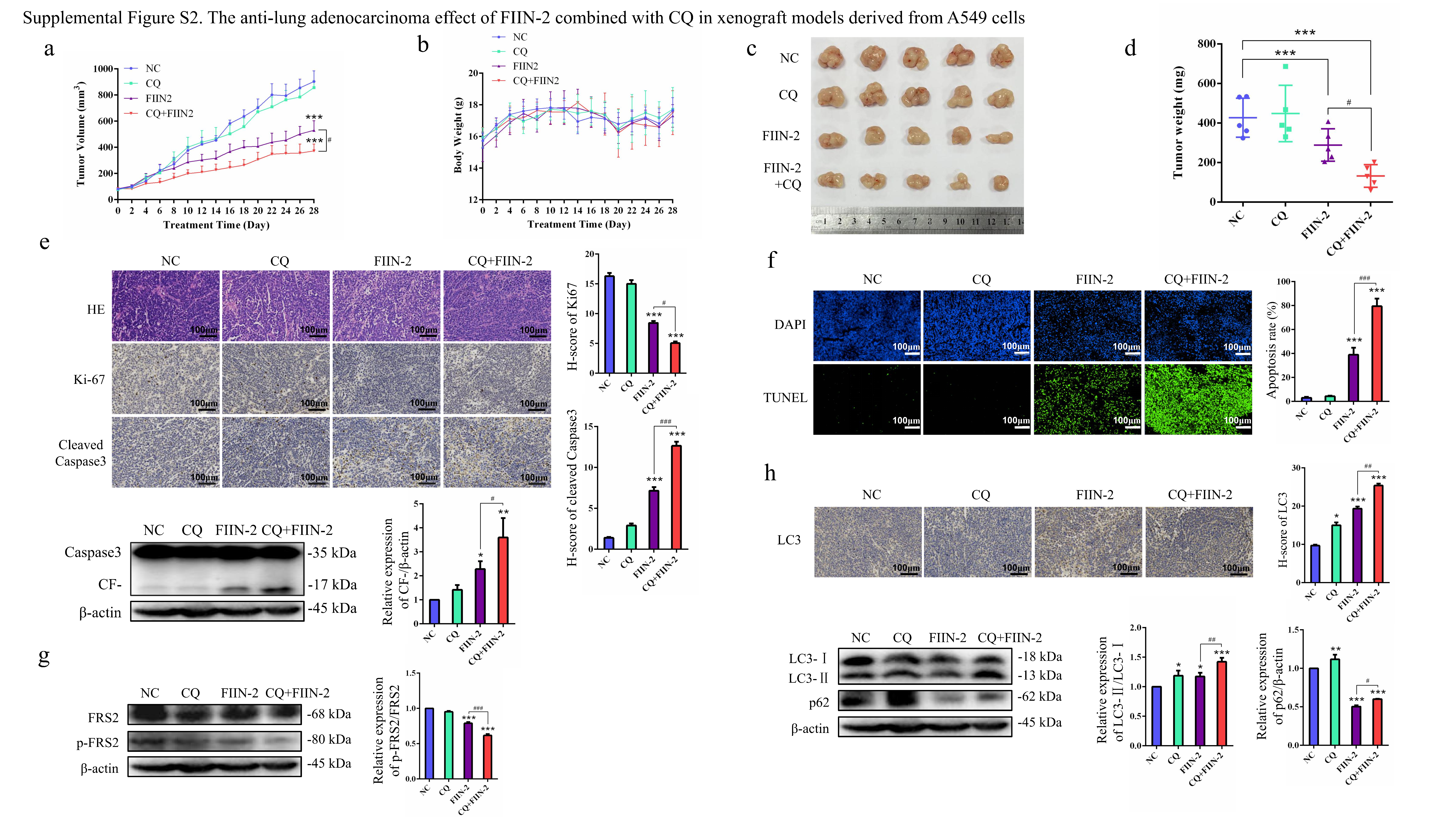

Supplement: Supplementary file 3 — Supplemental Figure S2. [file 41419_2022_5201_MOESM3_ESM.png]
