# Supplementary material for: Inhibition of autophagy potentiates the cytotoxicity of the irreversible FGFR1-4 inhibitor FIIN-2 on lung adenocarcinoma
Source: Cell Death Dis. 2022 Aug 30;13(8):750. doi: 10.1038/s41419-022-05201-0 (PMC9428205; doi:10.1038/s41419-022-05201-0)
Supplement: Supplementary file 4 — Supplemental Table S1. [file 41419_2022_5201_MOESM4_ESM.docx]

**Supplemental Table S1. Primers used for PCR**

| Target segment | Universal primers | Design sequence (5′ - 3′) | Length | Annealing temperature |
| --- | --- | --- | --- | --- |
| FGFR1 | FGFR1（F） | TACTCCCCCAGCTTTCCC | 18 bp | 57.1℃ |
|  | FGFR1（R） | AGACGGAATCCTCCCCTG | 18 bp | 56.8℃ |
| FGFR2 | FGFR2（F） | GGTGGCTGAAAAACGGGAAG | 20 bp | 57.2℃ |
|  | FGFR2（R） | AGATGGGACCACACTTTCCATA | 22 bp | 55.4℃ |
| FGFR3 | FGFR3（F） | TTAACACTTCTTACGCAATGCT | 22bp | 51.7℃ |
|  | FGFR3（R） | GCCCAGTAACAGTACAGAACGA | 22bp | 56.7℃ |
| FGFR4 | FGFR4（F） | GGTGACTCCTTCACCTCCA | 19bp | 56.8℃ |
|  | FGFR4（R） | AGCGGAACTTGACGGTGT | 18bp | 57.2℃ |
| Actin | Actin（F） | TGCCATCCTAAAAGCCAC | 18bp | 52.5℃ |
|  | Actin（R） | TCAACTGGTCTCAAGTCAGTG | 21bp | 54.2℃ |
