# Supplementary material for: Inhibition of autophagy potentiates the cytotoxicity of the irreversible FGFR1-4 inhibitor FIIN-2 on lung adenocarcinoma
Source: Cell Death Dis. 2022 Aug 30;13(8):750. doi: 10.1038/s41419-022-05201-0 (PMC9428205; doi:10.1038/s41419-022-05201-0)
Supplement: Supplementary file 5 — Supplemental Table S2. [file 41419_2022_5201_MOESM5_ESM.docx]

**Supplemental Table S2. The immunohistochemical expression of FGFR2 in lung adenocarcinoma**

|  | N  H-score* (‾x ± SD) P |
| --- | --- |
| FGFR2 | 19 7.79±3.19 |
| Low | 10 (52.63%) 5.20±1.03 ＜0.01 |
| High | 9 (47.37%) 10.67±2.00 |

* H-score: Low, 0-7; high, 8-12.
