# Supplementary material for: Inhibition of autophagy potentiates the cytotoxicity of the irreversible FGFR1-4 inhibitor FIIN-2 on lung adenocarcinoma
Source: Cell Death Dis. 2022 Aug 30;13(8):750. doi: 10.1038/s41419-022-05201-0 (PMC9428205; doi:10.1038/s41419-022-05201-0)

Fig. 1c

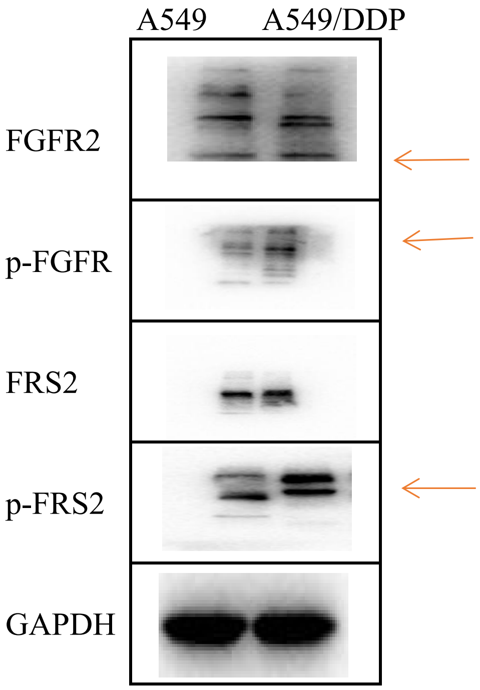

Fig. 1d

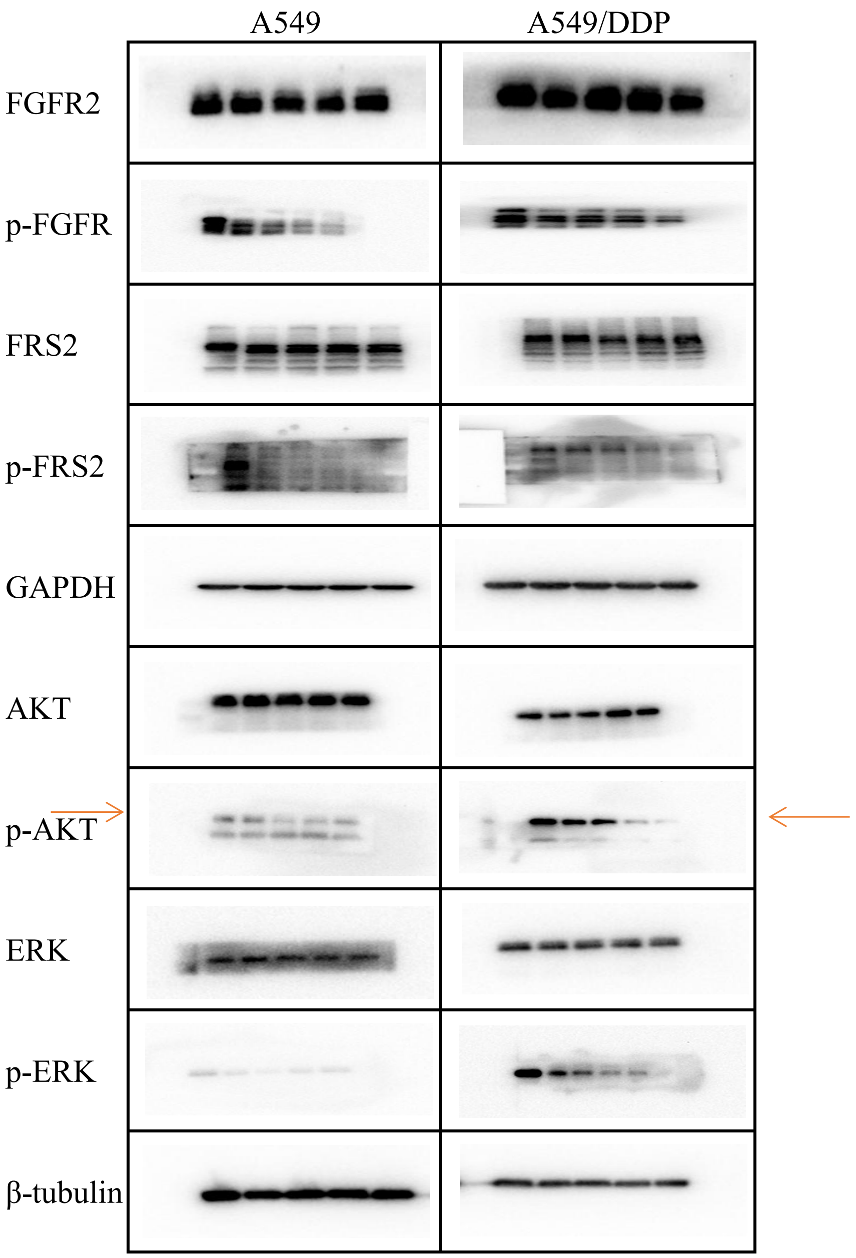
A549
A549/DDP
FGFR2
p-FGFR
FRS2
p-FRS2
GAPDH
AKT
p-AKT
ERK
p-ERK
β-tubulin

Fig. 2f

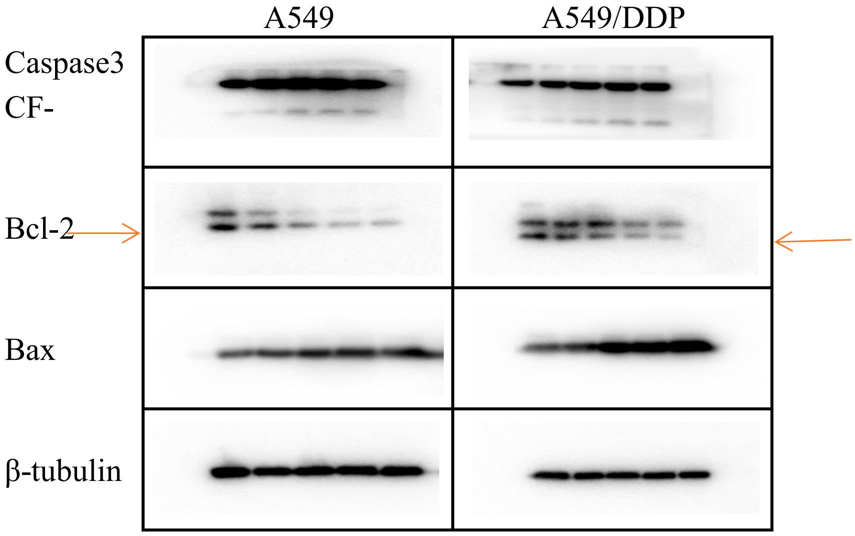

Fig. 3c

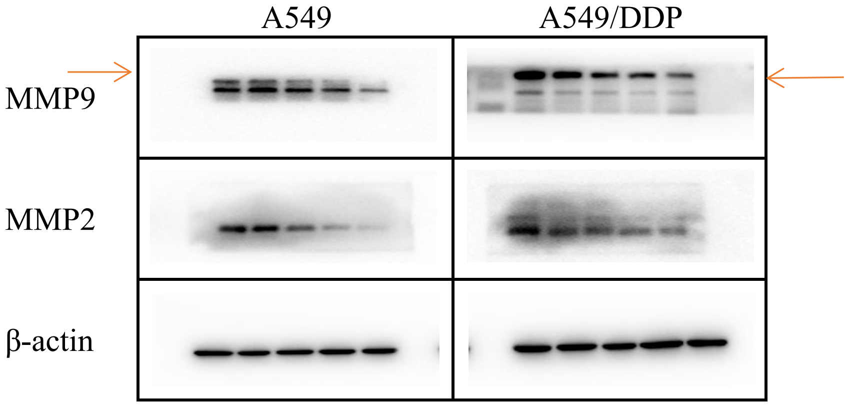

Fig. 4c

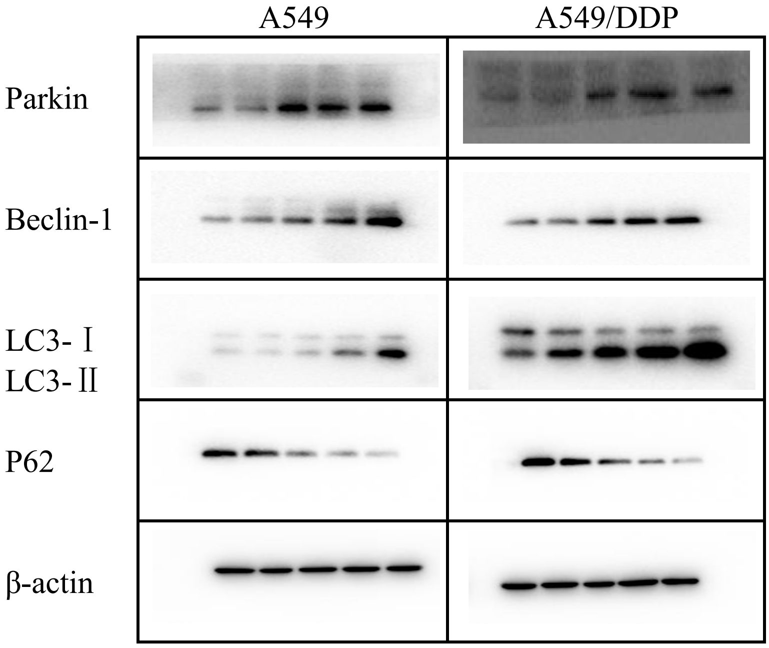

Fig. 4d

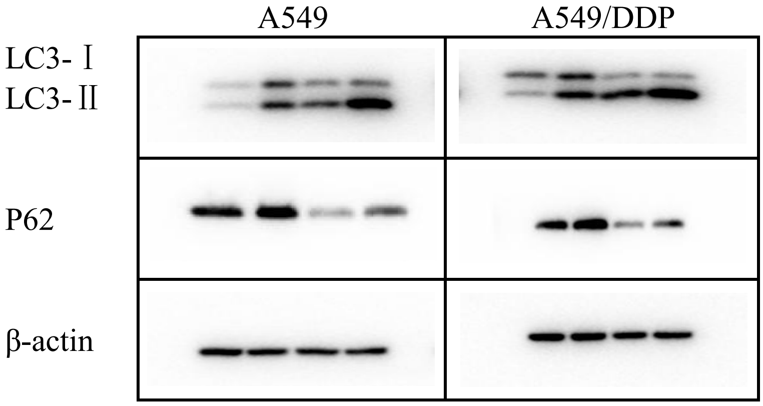

Fig. 4f

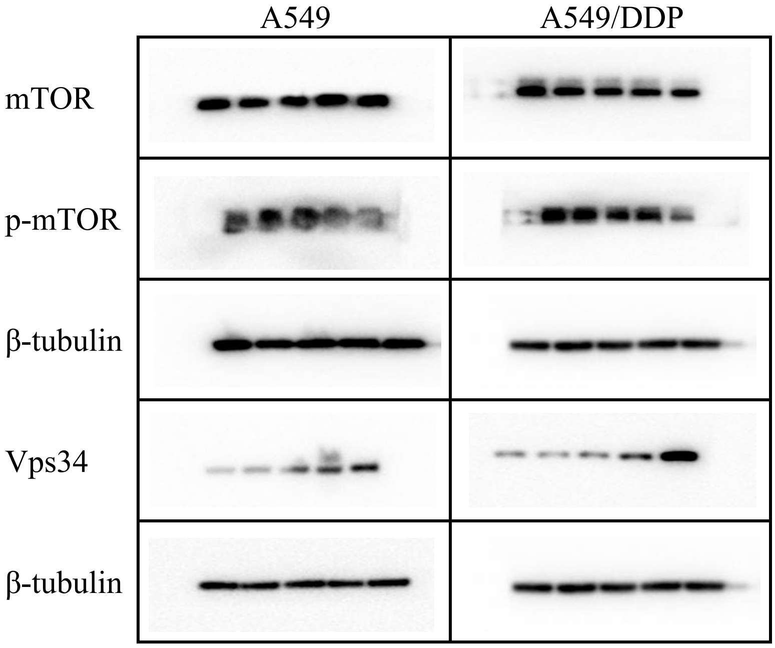

Fig. 4g

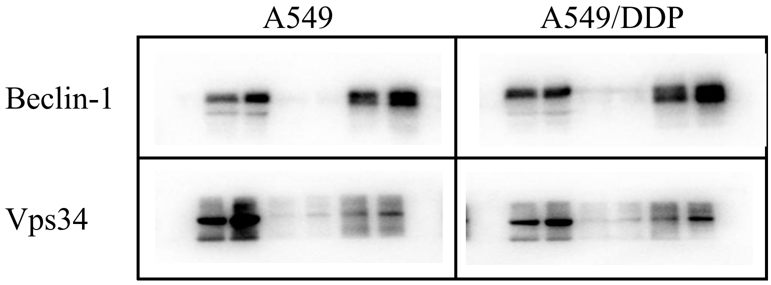

Fig. 5a

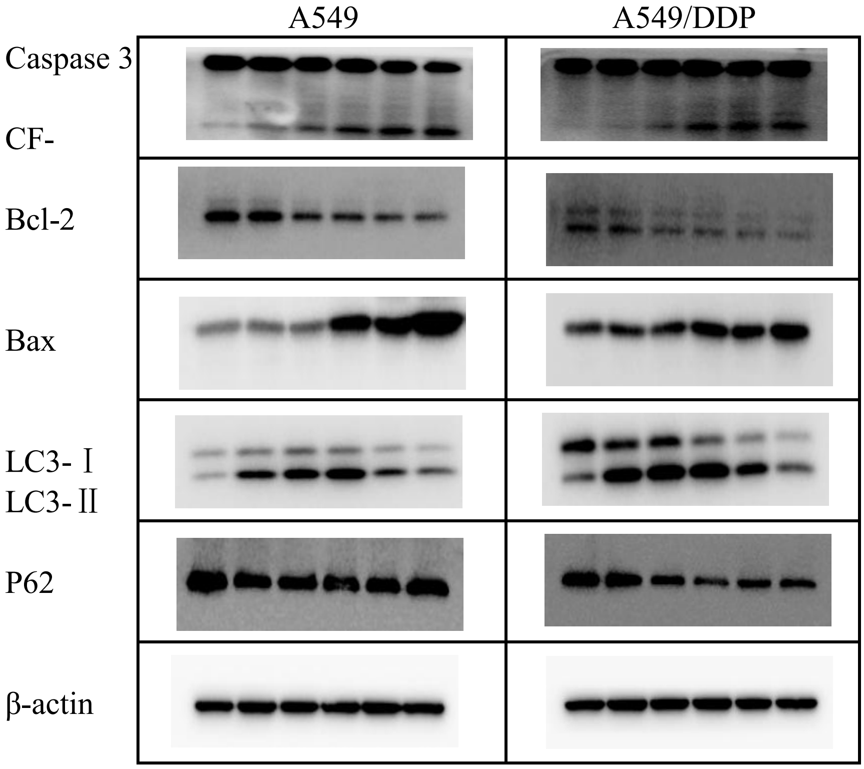

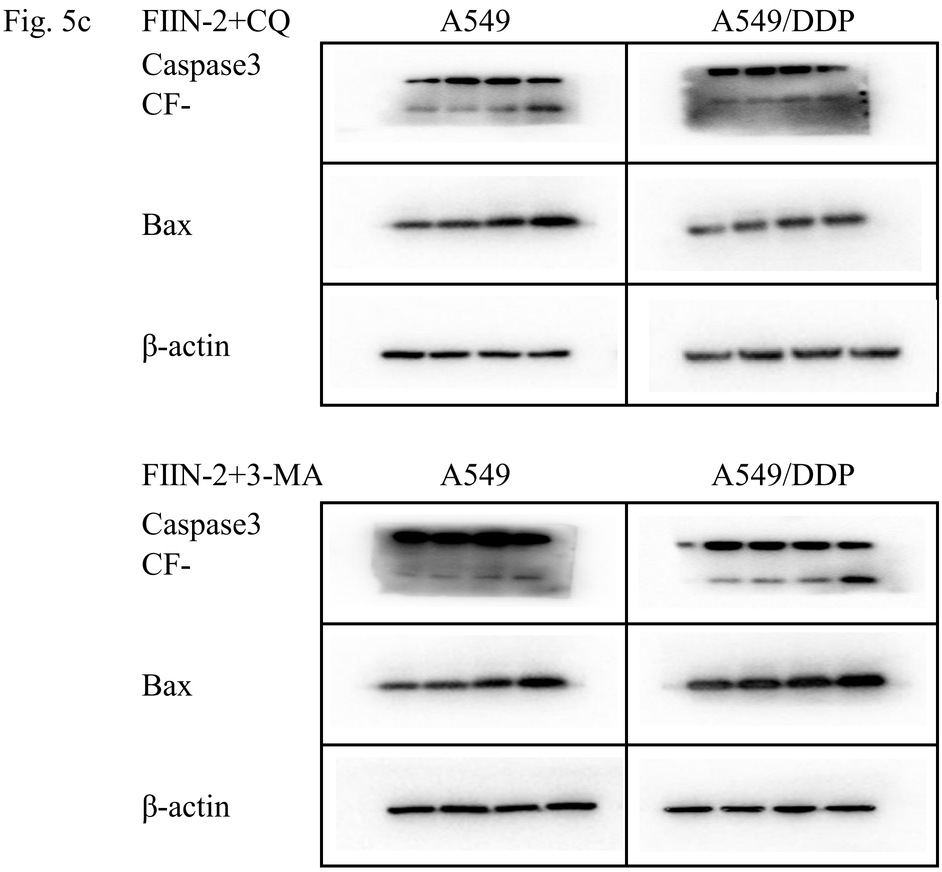
Fig. 5c
FIIN-2+CQ
A549
A549/DDP
Caspase3
CF-
Bax
β-actin
FIIN-2+3-MA
A549
A549/DDP
Caspase3
CF-
Bax
β-actin

Fig. 6e

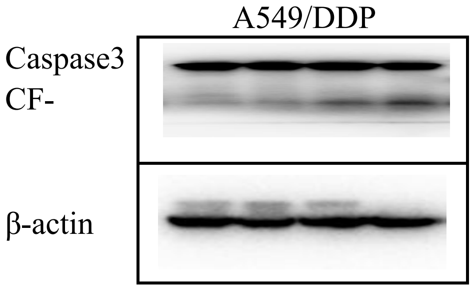

Fig. 6g

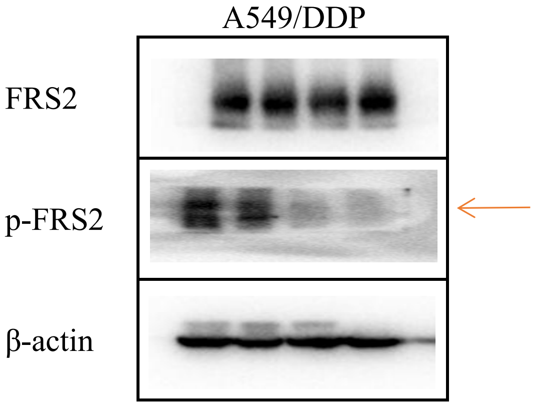

Fig. 6h

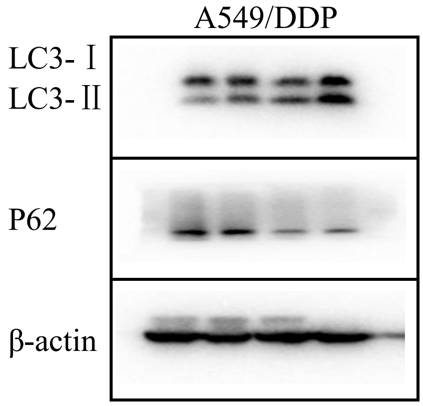

Supplemental Fig. S1

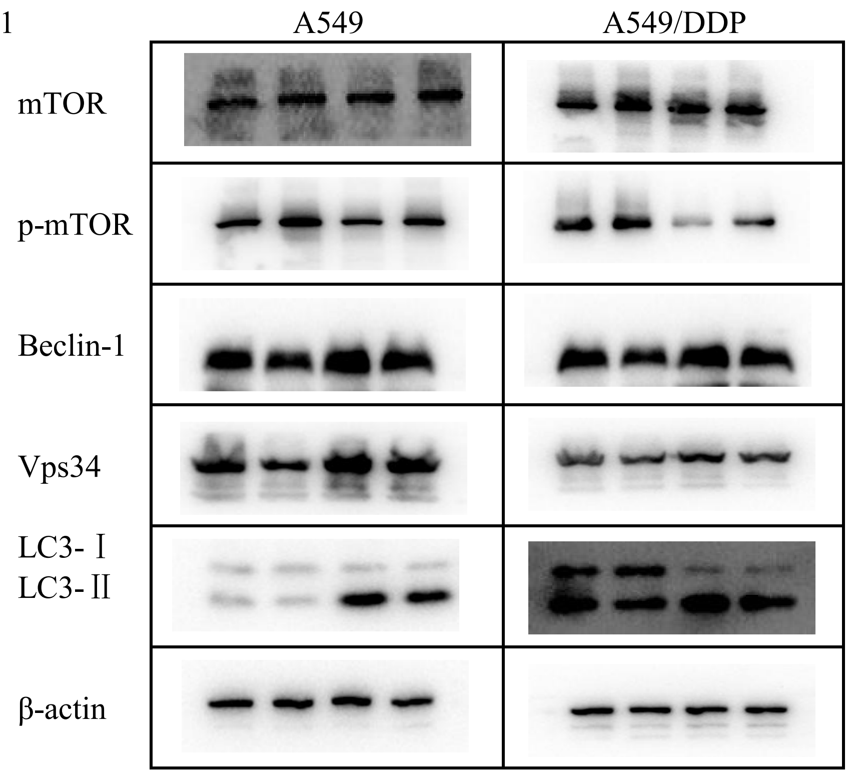

Supplemental Fig. S2e

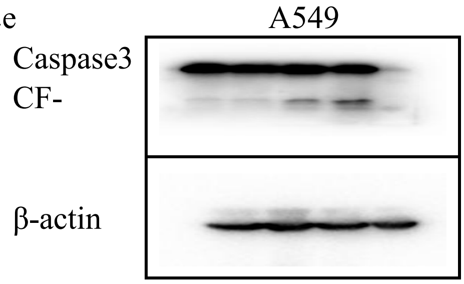

Supplemental Fig. S2g

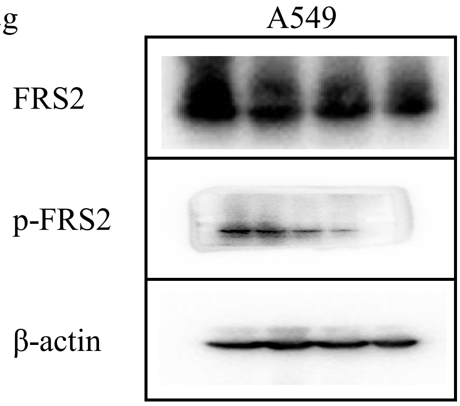

Supplemental Fig. S2h

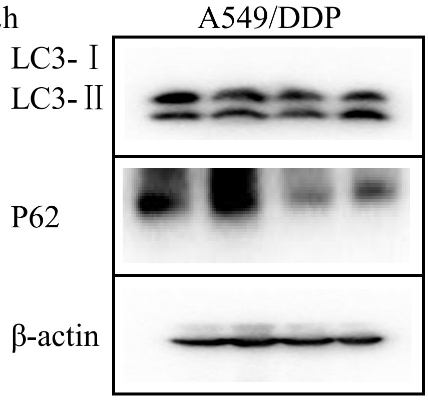

Supplement: Supplementary file 6 — Original full length western blots [file 41419_2022_5201_MOESM6_ESM.pdf]
